# Supplementary material for: Regulatory control of DNA end resection by Sae2 phosphorylation
Source: Nat Commun. 2018 Oct 1;9:4016. doi: 10.1038/s41467-018-06417-5 (PMC6167383; doi:10.1038/s41467-018-06417-5)
Supplement: Supplementary file 1 — Supplementary Information [file 41467_2018_6417_MOESM1_ESM.pdf]

Regulatory control of DNA end resection by Sae2 phosphorylation

Cannavo et al

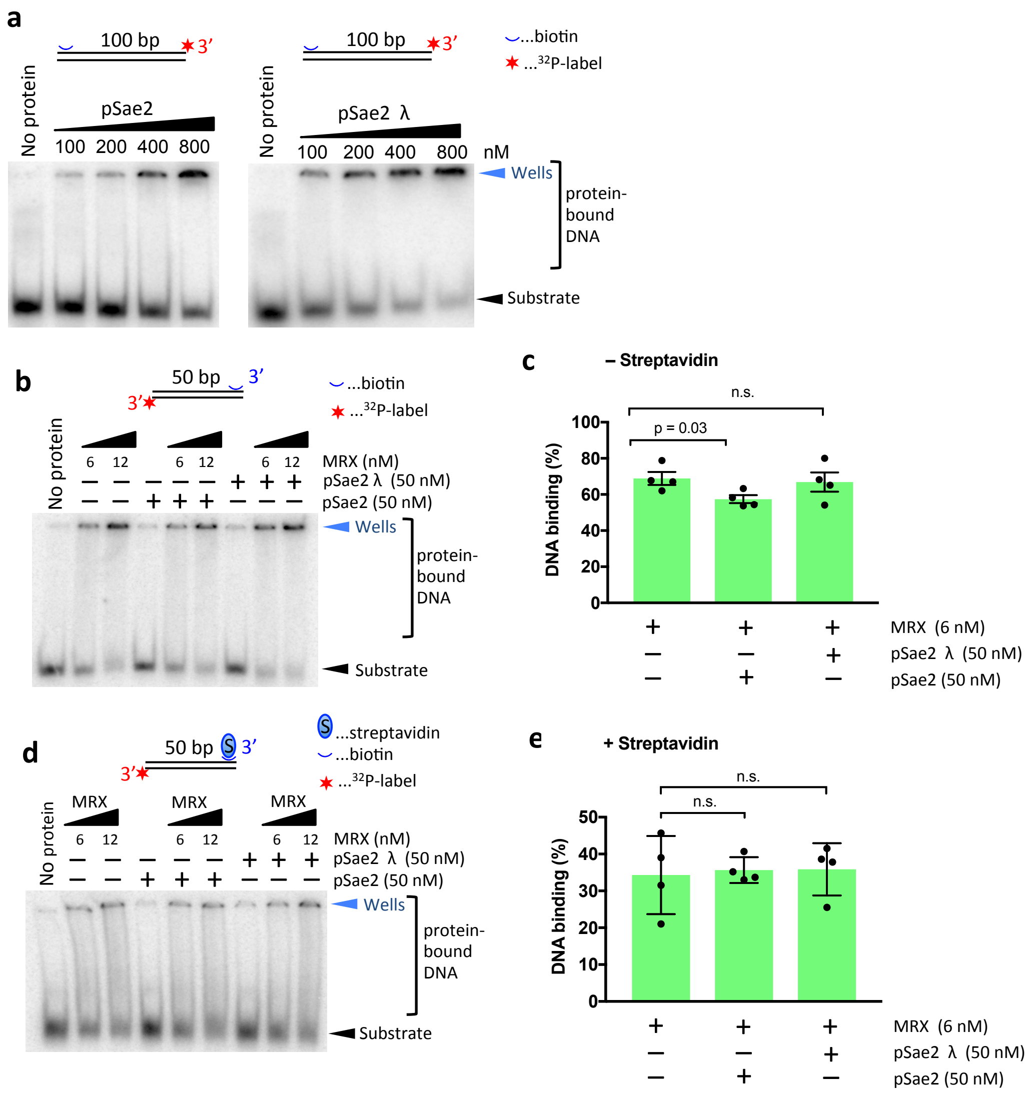

Supplementary Figure 1. Phosphorylation of Sae2 does not promote its DNA binding capacity. a, Representative electrophoretic mobility shift assay (6% polyacrylamide) with recombinant phosphorylated Sae2 (pSae2) or Sae2 dephosphorylated with λ phosphatase (pSae2 λ) and 100 bp-long dsDNA substrate. Position of gel wells is indicated. The reaction was incubated for 30 min at 30°C. **b**, Representative electrophoretic mobility shift assay with MRX, phosphorylated Sae2 (pSae2) and λ phosphatase-treated Sae2 (pSae2 λ), as indicated, and 50 bp-long dsDNA substrate with a biotin label but no streptavidin block. The reaction buffer contained ATP, and the reactions were incubated for 5 min at 30°C. The products were separated on a 6% native polyacrylamide gel. **c**, Quantitation of experiments such as shown in panel **b**, with 6 nM MRX. Error bars, SEM; n=4. **d**, Representative electrophoretic mobility shift assay with MRX, phosphorylated Sae2 (pSae2) and λ phosphatase-treated pSae2 (pSae2 λ), and 50 bp-long dsDNA substrate with one end blocked by streptavidin. The reaction buffer contained ATP, and the reactions were incubated for 30 min at 30°C. The products were separated on a 4% native polyacrylamide gel. **e**, Quantitation of experiments such as shown in panel **d**, with 6 nM MRX. Error bars, SEM; n=4.

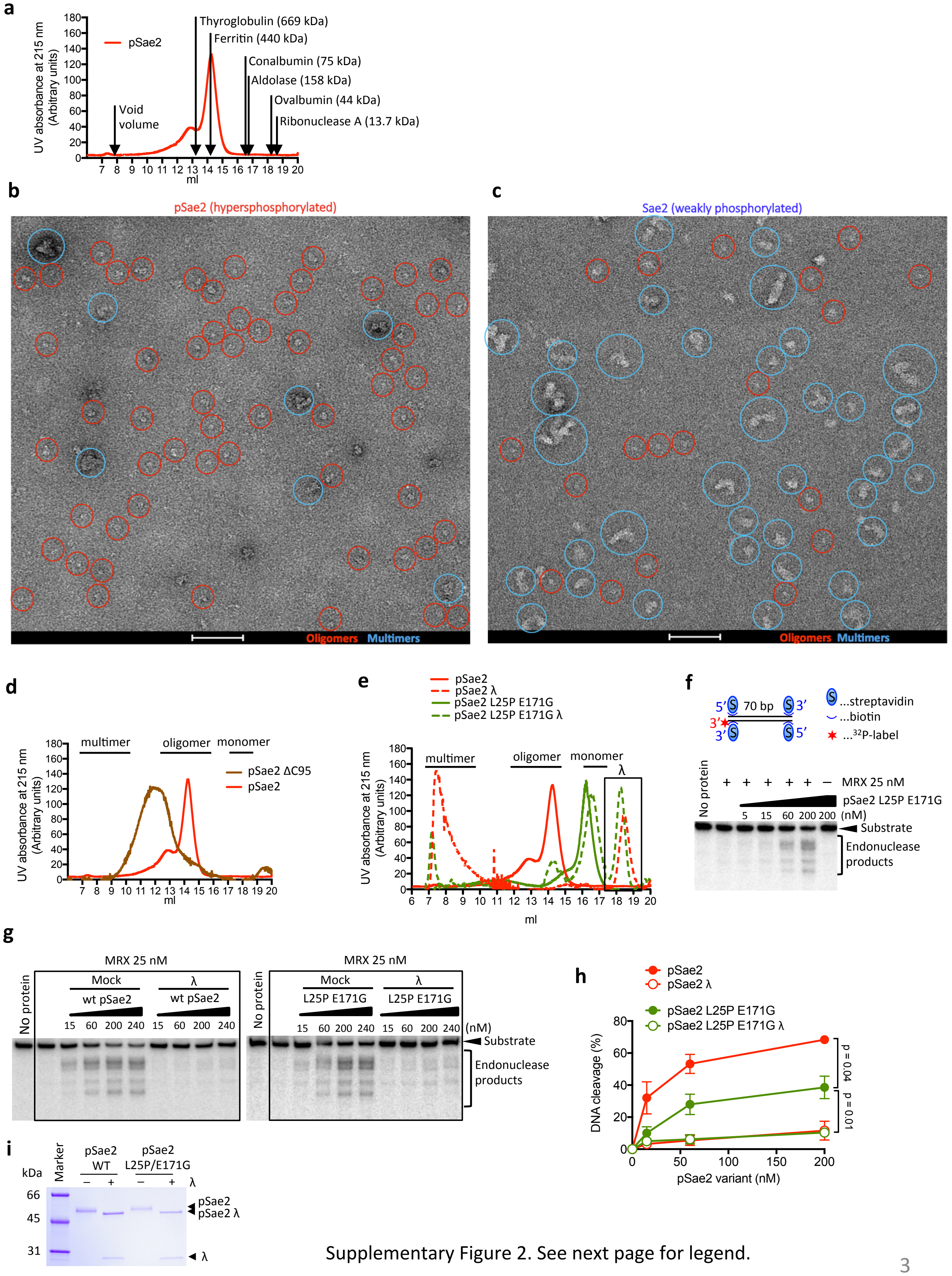

Supplementary Figure 2. See next page for legend.

Supplementary Figure 2. Sae2 oligomerization is important for its stimulatory effect on the MRX nuclease. **a**, Size exclusion chromatography with marker globular proteins. The profile of pSae2 is the same as in Fig. 2a, and is shown again for reference. **b**,**c**, Representative transmission electron micrographs of negatively stained Sae2 prepared with phosphatase inhibitors (pSae2, 800 nM, **b**) and without phosphatase inhibitors (Sae2, 800 nM, **c**). As these were taken closer to focus and in thinner stain than the micrographs in Fig. 1i,j, they more readily visualize the size distribution of smaller oligomers in pSae2 in comparison to Sae2. Sae2 oligomers are circled in red, larger multimers are circled in blue. Scale bar indicates 100 nm. **d**, Size exclusion chromatography analysis of the N-terminal fragment of pSae2 (pSae2 ΔC95, lacking 95 residues from the C-terminus), prepared in the presence of phosphatase inhibitors. The profile of pSae2 is the same as in Fig. 2a, and is shown again for reference. **e**, Size exclusion chromatography analysis of pSae2 L25P E171G, either mock-treated or λ phosphatase-treated, as indicated. Phosphorylated and dephosphorylated wild type Sae2 (pSae2 and pSae2 λ) are the same as in Fig. 1h, and are shown again for reference. The species indicated in the rectangle corresponds to the λ phosphatase protein. **f**, A representative nuclease assay with phosphorylated pSae2 L25P E171G, prepared with phosphatase inhibitors, and MRX, using a dsDNA substrate with both ends blocked with streptavidin. **g**, Representative nuclease assay with MRX and wild type pSae2 or pSae2 L25P E171G. The pSae2 variants were either mock-treated or λ phosphatase-treated, as indicated. **h**, Quantitation of experiments such as shown in panel **g**. Error bars, SEM; n≥3. The results shown for wt pSae2 are the same as in Fig. 3a, and are shown here for reference. **i**, Representative polyacrylamide gel electrophoresis of pSae2 or pSae2 L25P E171G, treated or not with λ phosphatase. The 4-15% polyacrylamide gradient gel was stained with Coomassie blue.

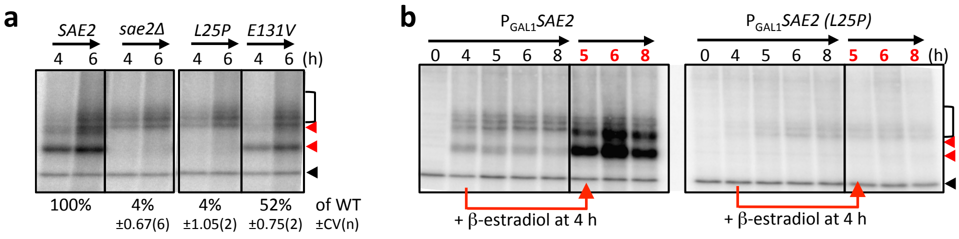

Supplementary Figure 3. Sae2 oligomerization is important for its activity *in vivo*. Spo11-oligonucleotide formation assay. **a**, *sae2*Δ cells were complemented with wt and *sae2 L25P* expressed from a centromeric vector with native promoter and terminator. Quantitation is shown below the lanes as an average (relative to wild type) ± coefficient of variation. **b**, *sae2*Δ cells were complemented with wt *SAE2* or *sae2 L25P* induced with β-estradiol 4 h after the onset of meiosis (in red). Non-induced samples are labeled in black. The red triangles mark the long and short Spo11-oligonucleotide species generated in wild type cells. The open bracket marks the double-cut Spo11-oligonucleotide species seen in wild type and *sae2Δ.* The black triangle marks a non-specific terminal deoxynucleotidyl transferase band.

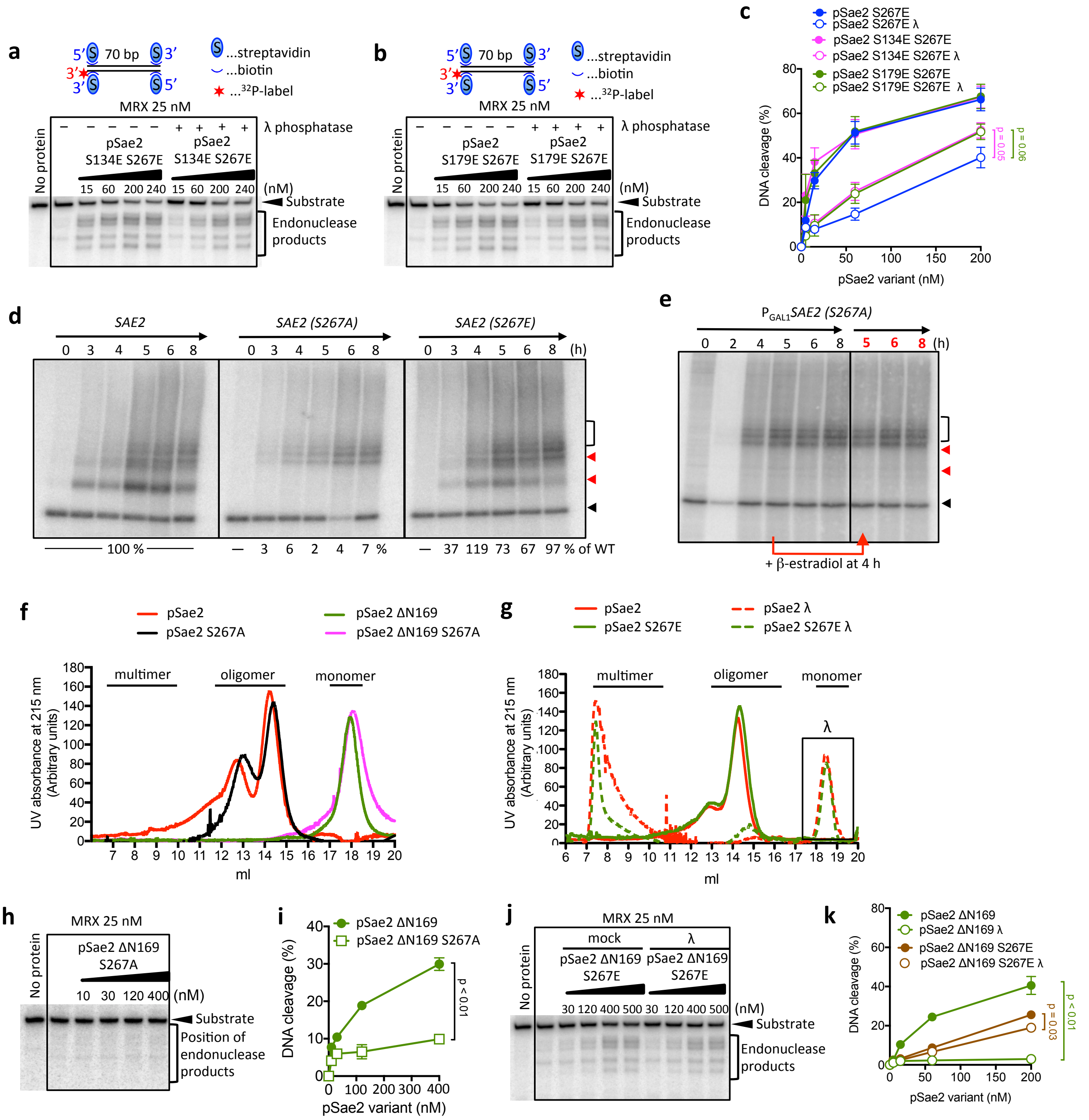

Supplementary Figure 4. CDK-dependent phosphorylation of Sae2 has a role in its DNA end resection capacity. a, A representative nuclease assay with MRX and pSae2 S134E S267E treated or not with λ phosphatase. **b**, A representative nuclease assay with MRX and pSae2 S179E S267E treated or not with λ phosphatase. **c**, Quantitation of experiments such as shown in panels **a** and **b**. The pSae2 S267E variant data is the same as in Fig. 3d, and is shown again for reference. Error bars, SEM; n=3. **d-e**, Spo11-oligonucleotide formation assay in *sae2*Δ cells with **d**, wt Sae2, Sae2 S267A and S267E expressed from a centromeric vector from its native promoter (quantitation is shown below the lanes as an average relative to wild type), or **e**, with Sae2 S267A expressed upon adding β-estradiol 4 h after the onset of meiosis (in red), with non-induced samples labeled in black. The red triangles mark the long and short Spo11-oligonucleotide species generated in wild type cells. The open bracket marks the MRX-Sae2 independent double-cut Spo11-oligonucleotide species. The black triangle marks a non-specific terminal deoxynucleotidyl transferase band.

Continued on next page.

Supplementary Figure 4. CDK-dependent phosphorylation of Sae2 has a role in its DNA end resection capacity.

Continued from previous page.

f, Size exclusion chromatography analysis of the indicated pSae2 variants, prepared in the presence of phosphatase inhibitors. The pSae2 ΔN169 dataset is identical to that shown in Fig. 2a, and is shown again for reference. **g**, Size exclusion chromatography analysis of the indicated pSae2 variants, prepared in the presence of phosphatase inhibitors, mock-treated or treated with λ phosphatase. The pSae2 and pSae2 λ datasets are identical to those shown in Fig. 1h, and are shown again for reference. The species in the rectangle corresponds to λ phosphatase. **h**, A representative nuclease assay with MRX and pSae2 ΔN169 S267A. The numbering corresponds to amino acid position in the full-length polypeptide. **i**, Quantitation of experiments such as shown in panel **h**. Error bars, SEM; n=3. **j**, A representative nuclease assay with MRX and pSae2 ΔN169 S267E. The numbering corresponds to amino acid position in the full-length polypeptide. **k**, Quantitation of experiments such as shown in panel **j**. Error bars, SEM; n≥4. The pSae2 ΔN169 data is the same as in Fig. 2c, and is shown again for reference.

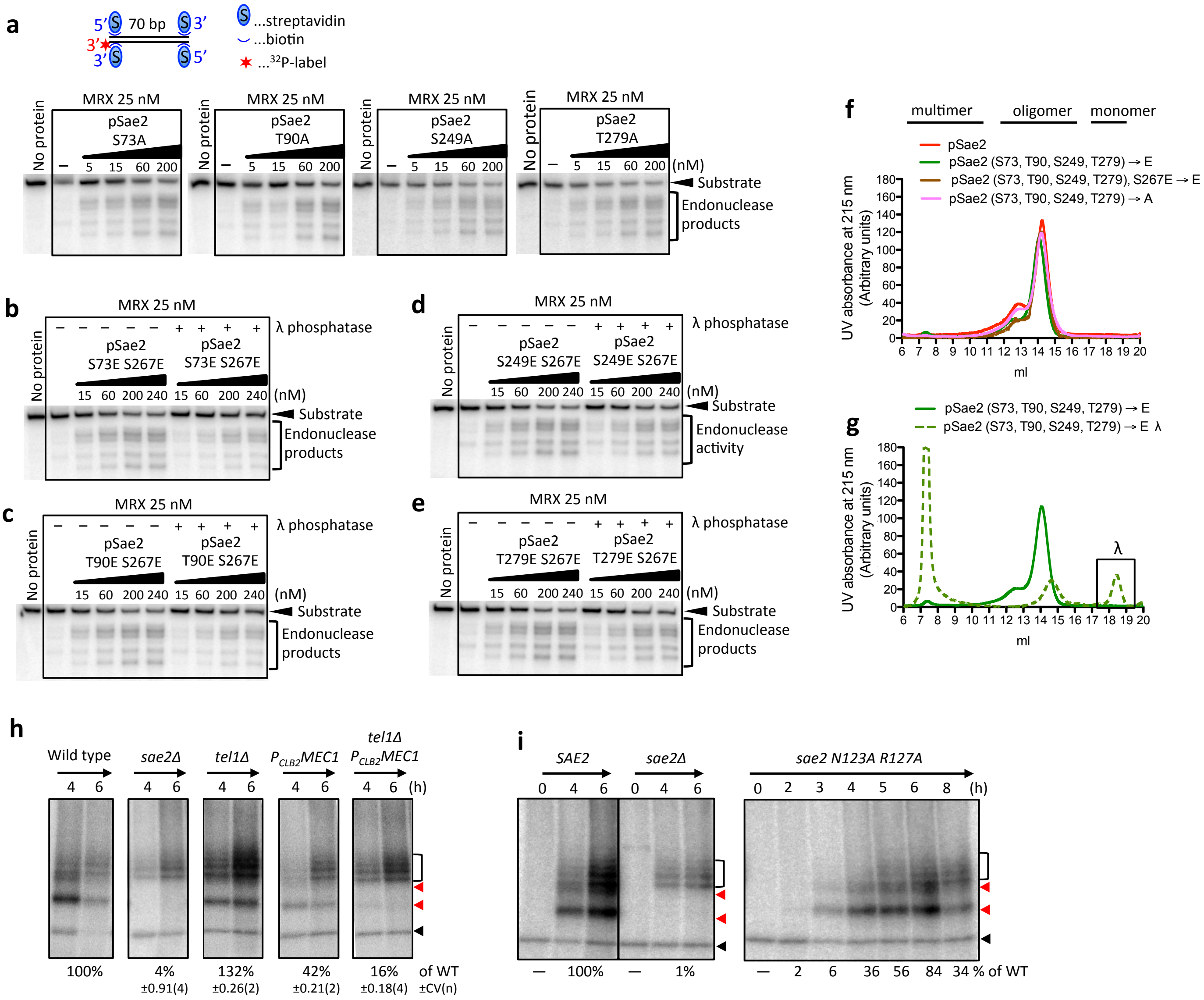

Supplementary Figure 5. The role of Mec1/Tel1-dependent phosphorylation of Sae2 on its DNA end resection capacity. a, Representative nuclease assays with the indicated pSae2 variants and MRX. **b-e**, Representative nuclease assays with the indicated pSae2 variants mock-treated or treated with λ phosphatase. **f**, Size exclusion chromatography analysis of the indicated pSae2 variants. The pSae2 dataset is identical to that shown in Fig. 2a, and is shown again for reference. **g**, Size exclusion chromatography analysis of the indicated pSae2 variants treated or mock-treated with λ phosphatase. The species in the rectangle corresponds to λ phosphatase. **h**, Spo11-oligonucleotide formation assay in wt, *tel1*Δ or *CLB2-MEC1* cells. Quantitation is shown below the lanes as an average relative to wild type ± coefficient of variation. **i**, Spo11-oligonucleotide formation assay in *sae2*Δ cells with wt *SAE2*, or *sae2 N123A R127A* expressed from a centromeric vector from its native promoter. Quantitation is shown below the lanes as an average relative to wild type. The red triangles mark the long and short Spo11-oligonucleotide species generated in wild type cells. The open bracket marks the MRX-Sae2-independent double-cut Spo11-oligonucleotide species. Black triangle marks a non-specific terminal deoxynucleotidyl transferase band.

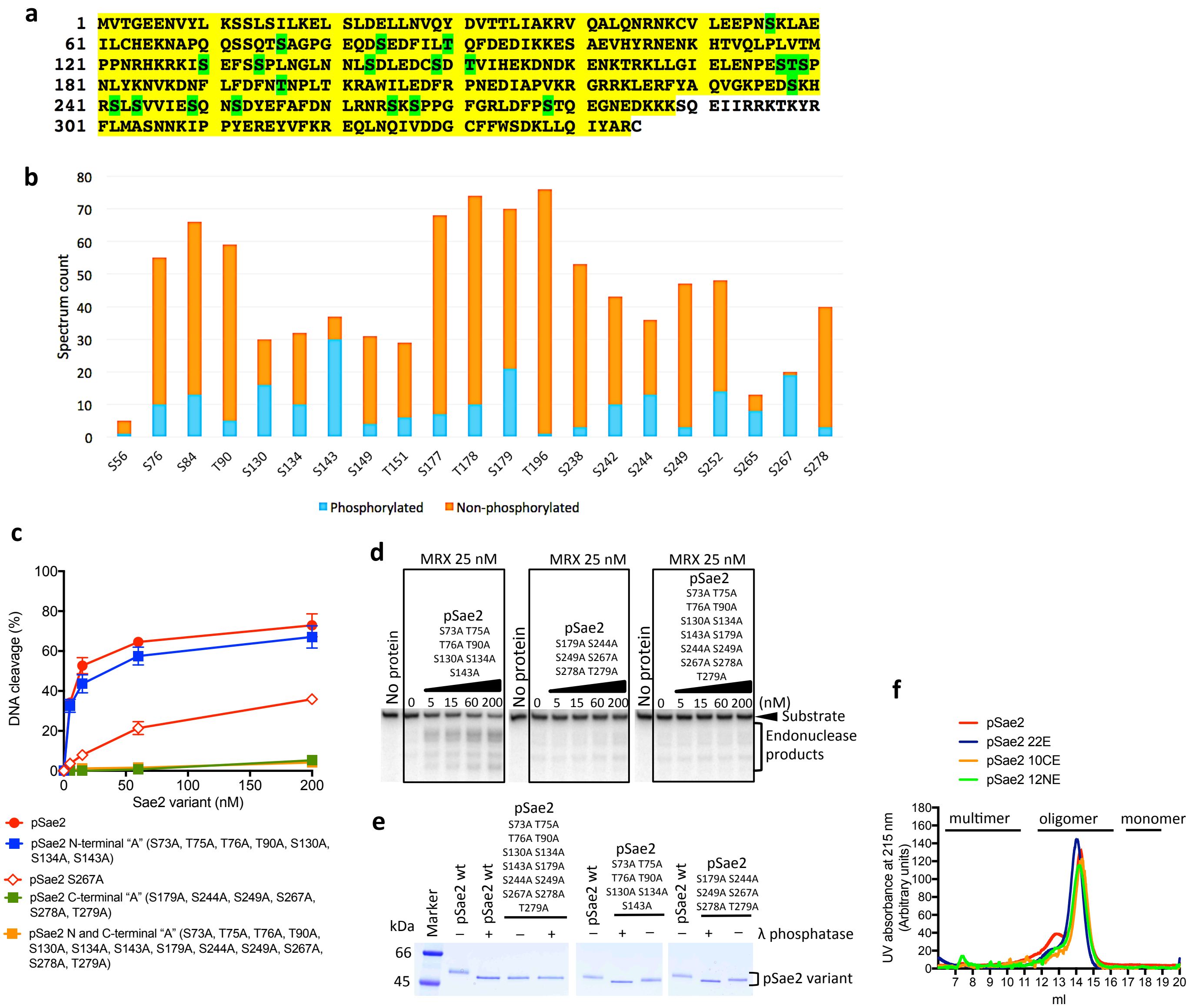

Supplementary Figure 6. Phosphorylation of Sae2 at its C-terminal, rather than the N-terminal part is important for its function to promote the MRX nuclease. a, Mass spectrometry analysis of phosphorylated recombinant pSae2. The sequence coverage is indicated in yellow and phosphorylated residues are indicated in green if they were identified with a site probability of at least 99%. **b**, Peptides of Sae2 covering individual phosphorylation sites were counted if the phosphorylation site probability was above 80% and the ratio between phosphorylated vs. non-phosphorylated is plotted. **c**, Quantitation of nuclease assays with MRX (25 nM) and indicated concentrations of the respective pSae2 variants. Error bars, SEM; n≥4. **d**, Representative nuclease assays with MRX and indicated pSae2 variants. **e**, Coomassie-stained polyacrylamide gels (4-15%) showing the pSae2 variants used in experiments in panels **c** and **d**, treated or not with λ phosphatase. **f**, Size exclusion chromatography analysis of the indicated pSae2 variants. The pSae2 dataset is identical to that shown in Fig. 2a, and pSae2 22E as in Fig. 5g, and are shown again for reference.

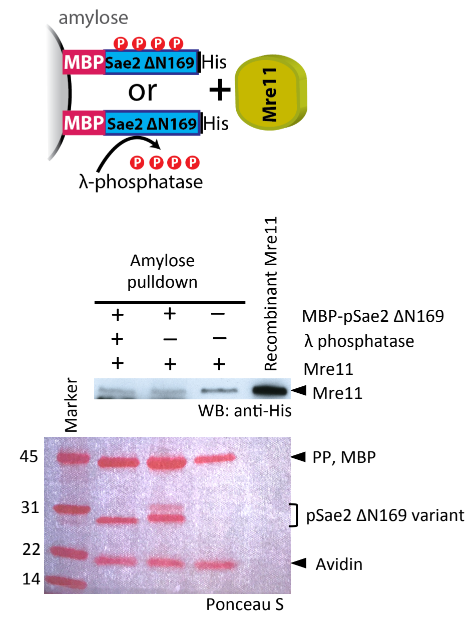

Supplementary Figure 7. Interaction of Sae2 and MRX. The phosphorylated recombinant MBP-tagged C-terminal domain of pSae2 (residues 170-345, pSae2 ΔN169) was bound to amylose resin, mock-treated or dephosphorylated with λ phosphatase, and incubated with recombinant Mre11. Proteins were eluted and visualized by Ponceau staining or Western blotting. Prescission protease (PP) was used to cleave MBP tag from MBP-pSae2 ΔN169, in order to facilitate detection of Mre11, which migrates above. Avidin was added to the elution buffer and shows equal loading. The C-terminal fragment of Sae2 does not interact with Mre11, irrespectively of phosphorylation.
